# Supplementary material for: Characterization of Oral Veillonella Species in Dental Biofilms in Healthy and Stunted Groups of Children Aged 6–7 Years in East Nusa Tenggara
Source: Int J Environ Res Public Health. 2022 Oct 27;19(21):13998. doi: 10.3390/ijerph192113998 (PMC9656475; doi:10.3390/ijerph192113998)
Supplement: Supplementary file 1 [file ijerph-19-13998-s001.zip › ijerph-1969985-supplementary.pdf]

Standar curves of 7 oral *Veillonella* spp.

a)

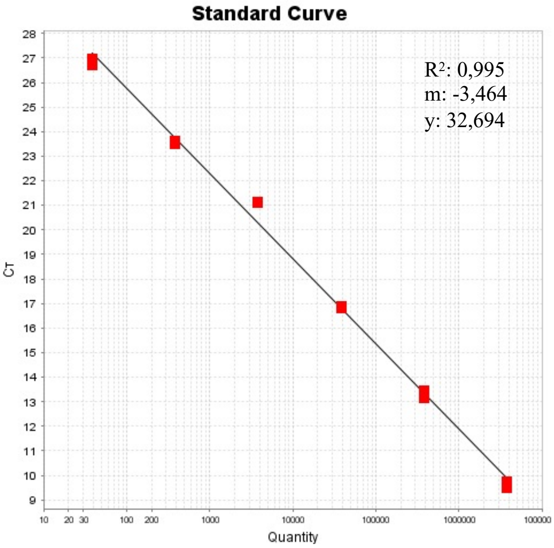

Veillonella atypica (ATCC 17744T)

b)

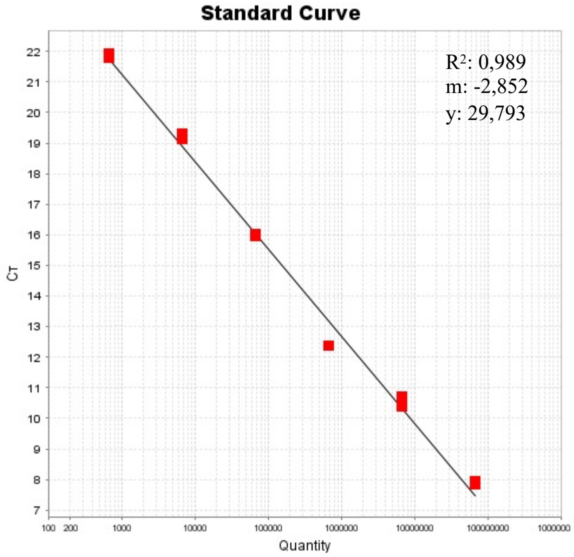

Veillonella denticariosi (ATCC 15641T)

c)

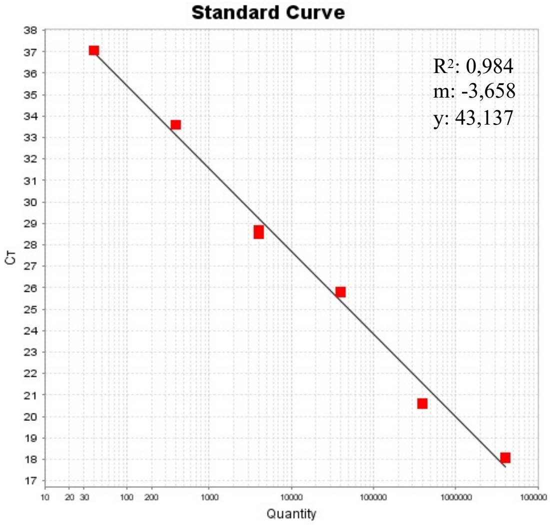

Veillonella dispar (ATCC 17748T)

d)

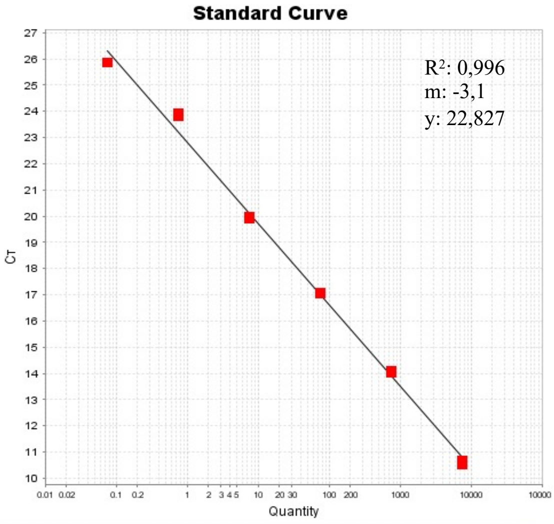

Veillonella infantium (JCM 31738T)

e)

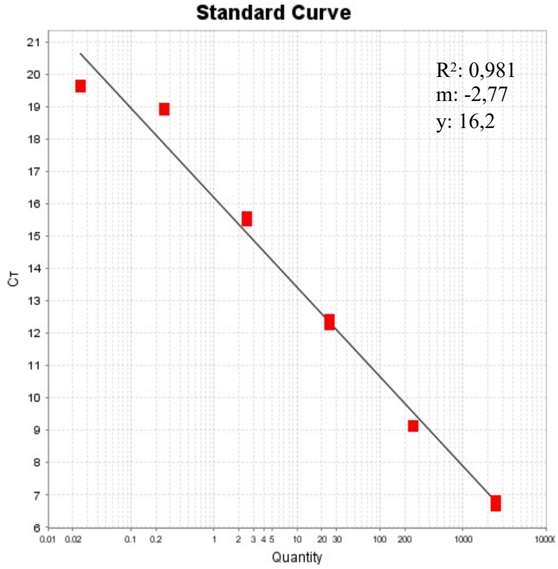

Veillonella parvula (ATCC 10790T)

f)

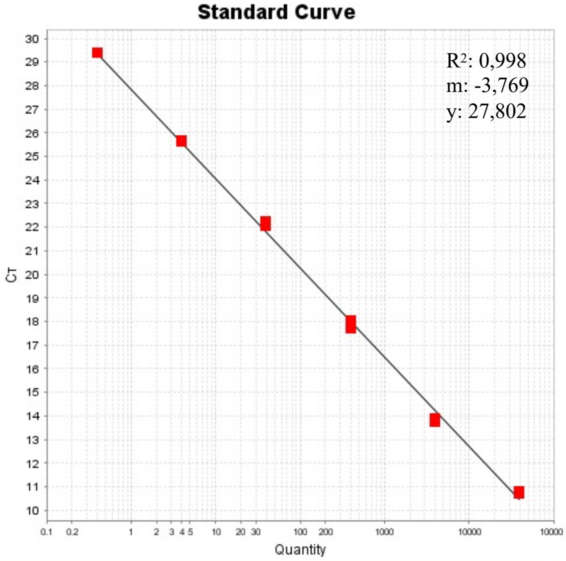

Veillonella rogosae (ATCC 15642T)

g)

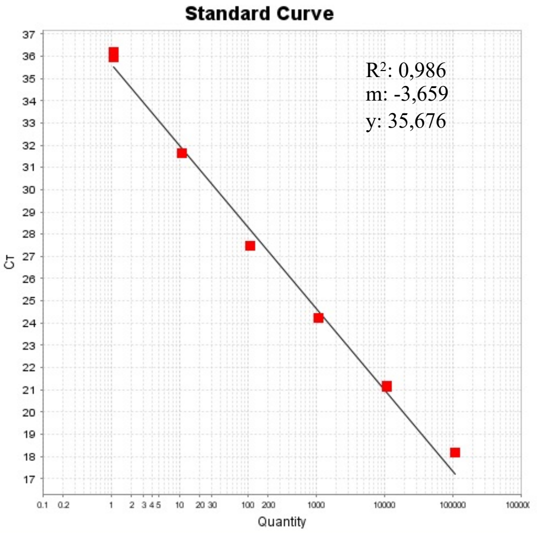

Veillonella tobetsuensis (ATCC 2400T)
